# Long-term psychological outcomes of flood survivors of hard-hit areas of the 1998 Dongting Lake flood in China: Prevalence and risk factors

**DOI:** 10.1371/journal.pone.0171557

**Published:** 2017-02-07

**Authors:** Wenjie Dai, Atipatsa C. Kaminga, Hongzhuan Tan, Jieru Wang, Zhiwei Lai, Xin Wu, Aizhong Liu

**Affiliations:** 1 Department of Epidemiology and Health Statistics, Xiangya School of Public Health, Central South University, Changsha, Hunan, China; 2 Department of Mathematics, Mzuzu University, Mzuzu, Malawi; 3 Department of Pediatrics, University of Pittsburgh School of Medicine, Pittsburgh, Pennsylvania, United States of America; 4 Immune Planning Division, Hunan Provincial Center for Disease Control and Prevention, Changsha, Hunan, China; Stellenbosch University, SOUTH AFRICA

## Abstract

**Background:**

Although numerous studies have indicated that exposure to natural disasters may increase survivors’ risk of post-traumatic stress disorder (PTSD) and anxiety, studies focusing on the long-term psychological outcomes of flood survivors are limited. Thus, this study aimed to estimate the prevalence of PTSD and anxiety among flood survivors 17 years after the 1998 Dongting Lake flood and to identify the risk factors for PTSD and anxiety.

**Methods:**

This cross-sectional study was conducted in December 2015, 17 years after the 1998 Dongting Lake flood. Survivors in hard-hit areas of the flood disaster were enrolled in this study using a stratified, systematic random sampling method. Well qualified investigators conducted face-to-face interviews with participants using the PTSD Checklist-Civilian version, the Zung Self-Rating Anxiety Scale, the Chinese version of the Social Support Rating Scale and the Revised Eysenck Personality Questionnaire-Short Scale for Chinese to assess PTSD, anxiety, social support and personality traits, respectively. Logistic regression analyses were used to identify factors associated with PTSD and anxiety.

**Results:**

A total of 325 participants were recruited in this study, and the prevalence of PTSD and anxiety was 9.5% and 9.2%, respectively. Multivariable logistic regression analyses indicated that female sex, experiencing at least three flood-related stressors, having a low level of social support, and having the trait of emotional instability were risk factors for long-term adverse psychological outcomes among flood survivors after the disaster.

**Conclusions:**

PTSD and anxiety were common long-term adverse psychological outcomes among flood survivors. Early and effective psychological interventions for flood survivors are needed to prevent the development of PTSD and anxiety in the long run after a flood, especially for individuals who are female, experience at least three flood-related stressors, have a low level of social support and have the trait of emotional instability.

## Introduction

Natural disasters, such as earthquakes, cyclones, floods and tsunamis, not only cause direct economic loss, death and physical injury but also lead to long-term adverse psychological outcomes [1, 2]. Of these adverse psychological outcomes, post-traumatic stress disorder (PTSD) is the most prevalent, followed by anxiety [3, 4]. For example, it has been reported that 13 to 14 months after the 2008 Wenchuan earthquake in China, the prevalence of PTSD and anxiety among the elderly was 26.3% and 42.9%, respectively [5], while six months after the same earthquake, these rates were 15.8% and 40.5% among adolescents [6]. Additionally, the prevalence of PTSD and anxiety was, respectively, 26.9% and 12.0%, in India 14 months after the super-cyclone Orissa [7]; 22% and 48% in England 3 to 6 months after the 2007 summer floods [8]; and 16.8% and 17.8% two and a half years after the 2004 Indian Ocean tsunami among Swiss tourists who had been affected by the event [9]. The differences in the observed prevalence of PTSD and anxiety across these studies may be attributed to the type of trauma, degree of exposure to trauma, characteristics of the selected samples, the specific measurements applied to identify PTSD and anxiety, and the time gap from the trauma to the assessment of PTSD and anxiety [10, 11].

Numerous studies have shown that long-term adverse psychological outcomes may lead to a decreased quality of life, as well as impaired cognitive and social functioning [12–15]. Therefore, reducing the prevalence of long-term adverse psychological outcomes is necessary and could be achieved by providing effective psychological intervention services to individuals with a higher risk of suffering from these consequences [16]. Thus, early identification of the risk factors for long-term adverse psychological outcomes is essential because it could contribute substantially to identifying individuals who are at an increased risk of experiencing these outcomes [12, 17]. Currently, research has shown that female sex, being older, having more severe exposure to a disaster, having a low level of social support and having the trait of neuroticism are related to long-term adverse psychological outcomes [12, 16, 18–20]. In China, most research exploring long-term psychological outcomes among survivors following natural disasters has focused on the 2008 Wenchuan earthquake. However, studies on the long-term psychological outcomes of flood survivors are limited, despite the fact that China has been plagued by floods for many decades [21–23].

A devastating flood struck Yangzi River in China in 1998, causing 4,150 deaths, destroying 6.9 million houses and displacing 18.4 million people [24]. Dongting Lake, located in Hunan Province, was the area most affected by the flood. The flood not only made hundreds of thousands of survivors homeless but also left some survivors suffering from mental health problems [25, 26]. The estimated prevalence of PTSD among the Dongting Lake survivors of this flood, for example, ranged from 8.6% to 9.2% two years after the flood [25, 26]. Moreover, a previous study conducted by our research group found that 15.89% of the 1998 Dongting Lake flood survivors who met the diagnostic criteria for PTSD in 2000 continued to suffer from PTSD in 2013–2014 and that social support was associated with recovery from PTSD [27]. However, the long-term psychological outcomes, especially symptoms of anxiety, among survivors of that flood disaster overall, not limited to survivors with PTSD in 2000, remain unknown. Thus, it was hypothesized in this study that although 17 years had passed since the Dongting Lake flood in 1998, PTSD and anxiety would both be prevalent among the survivors and that the prevalence of PTSD and anxiety would be related to certain socio-demographic characteristics, the degree of exposure to the flood, social support and personality traits. Therefore, survivors from hard-hit areas of the 1998 Dongting Lake flood were enrolled in this study to explore the prevalence of PTSD and anxiety and to identify the risk factors for PTSD and anxiety in the long term after the flood.

## Materials and methods

### Ethics statement

This investigation was approved by the Ethics Committee of Xiangya School of Public Health, Central South University of China. Written informed consent was obtained from the participants.

### Participants and procedure

This cross-sectional study was conducted in December 2015, 17 years after the Dongting Lake flood in 1998. Huarong County, located in the south end of the middle reach of the Dongting Lake, which was directly exposed to the 1998 Dongting Lake flood, is a flood-prone area. Flood survivors in Huarong County were enrolled in this study using a stratified, systematic random sampling method. Firstly, Zhuzikou and Xingfu towns were randomly selected from the southeast of Harong County; these towns were next to Donting Lake and were hard-hit areas of the flood disaster in 1998. Then, using a systematic sampling approach, four villages from each town were selected. Finally, 30% of the total households in each selected village were enrolled in this study.

The inclusion criteria for participants in this study were as follows: (1) being at least 7 years old in 1998; (2) having experienced the 1998 Donting Lake flood; and (3) being willing to provide informed consent. Excluded from this study were individuals who (1) could not express themselves clearly, such as individuals with dementia, and (2) had received any psychological treatment since the 1998 Dongting Lake flood. Well qualified investigators conducted face-to-face interviews with participants to obtain information about socio-demographic characteristics, flood-related stressors, social support and personality traits and to identify PTSD and anxiety symptoms. Data collection procedures for this study were the same as those for our previous study [27].

### Measures

#### Flood-related stressors

Consistent with previous studies exploring psychological outcomes after natural disasters [5, 28], the degree of one’s exposure to the flood was measured by the total amount of flood-related stressors. This information was obtained by asking the following “Yes” or “No” questions: Have you lost at least one family member due to the 1998 Dongting Lake flood? Have you or your family members been physically injured due to the 1998 Dongting Lake flood? Have your houses been destroyed due to the 1998 Dongting Lake flood? Have you or your family lost most of your property due to the 1998 Dongting Lake flood? Have you or your family lost your livelihood due to the 1998 Dongting Lake flood? Individuals who answered “Yes” to exactly one question would be considered to experience one flood-related stressor. Thus, the total amount of flood-related stressors ranged from 0 to 5. Experiencing more flood-related stressors indicates more serious exposure to the flood.

#### Social support

Social support was assessed using the Chinese version of the Social Support Rating Scale (SSRS). This instrument comprises ten items that are each scored from 1 (none) to 4 (great). The total score of the ten items is used to assess individuals’ social support level, and this level is defined as low when the total score is from 12 to 44, medium when the total score is from 45 to 54, and high when the total score is greater than 55 [29]. The reliability and validity of this instrument have been shown to be high in a wide range of Chinese populations, with a Cronbach’s alpha coefficient equal to 0.91 [30, 31].

#### Personality traits

Personality traits were evaluated using the Revised Eysenck Personality Questionnaire-Short Scale for Chinese (EPQ-RSC). This instrument contains 4 subscales: extraversion, psychoticism, neuroticism and lie. Each subscale has 12 items with either “Yes” or “No” responses. The total score for each subscale was transformed according to the Chinese norm. For the extraversion subscale, extraversion is defined when the total score is greater than 56.7; middle is defined when the total score is from 43.3 to 56.7, and introversion is defined when the total score is less than 43.3. For the psychoticism subscale, a total score of less than 43.3 defines mild, while a total score from 43.3 to 56.7 defines middle, and a total score greater than 56.7 defines tough-minded. The total score for the neuroticism subscale indicates emotional stability when it is less than 43.3, middle when it is from 43.3 to 56.7 and emotional instability when it is greater than 56.7. Individuals with a transformed total score for the ‘lie’ subscale of 60 or greater were excluded, since the information they provided may not have been credible [32]. The EPQ-RSC has been extensively used among different populations in China and has demonstrated good reliability and validity, with Cronbach’s alpha coefficients greater than 0.70 with the exception of the psychoticism subscale [32, 33].

#### PTSD

PTSD was identified using the PTSD Checklist-Civilian version (PCL-C). The PCL-C is a self-report questionnaire with 17 items that correspond to the fourth edition of the Diagnostic and Statistical Manual (DSM-IV) criteria and is widely used when structured clinical interviews are not feasible [34]. The scale of each of these 17 items ranges from 1 (not at all) to 5 (extremely); thus, individuals’ total scores range from 17 to 85. An adult with a total score of 38 or greater is identified as having PTSD [35, 36]. In this study, the traumatic event in the original PCL-C was replaced by the 1998 Dongting Lake flood. The PCL-C has been widely used in a wide range of Chinese populations and has shown high internal consistency, with a Cronbach’s alpha coefficient equal to 0.96 [5, 37, 38].

#### Anxiety

Anxiety was identified using the Zung Self-Rating Anxiety Scale (SAS). The SAS is a self-report questionnaire with 20 items scored on a 4-point Likert scale ranging from 1 (never) to 4 (always). Thus, the total score of this instrument ranges from 20 to 80. Higher scores indicate more severe anxiety symptoms, and an adult with a total score of 50 or greater is identified as having anxiety [39, 40]. This instrument has been extensively used in China among different populations and has proven to be acceptable regarding its psychometric properties in previous studies [41, 42].

### Statistical analyses

Statistical analyses were performed using Statistical Package for the Social Sciences (SPSS) Version 19.0 software (IBM Corp, Armonk, NY). Frequencies and percentages (%) were presented to describe categorical data, and means with corresponding standard deviations (SDs) were used to describe continuous data. Univariable logistic regression analyses were conducted to investigate the bivariate associations between potentially associated variables (gender, age, education level, flood-related stressors, social support and personality traits) and PTSD and anxiety. Multivariable logistic regression analyses were used to identify the independent role of each of the associated factors for PTSD and anxiety [43]. Consistent with some previous studies [44, 45], factors with a *P* value less than or equal to 0.20 in the univariable logistic regression analyses were entered into the multivariable logistic regression analyses in case there were some important variables missing due to the limited sample size. The 95% confidence interval (95% CI) was presented for each odds ratio (OR). All statistical tests were two-tailed, and the significance level was set to be at most 0.05.

## Results

### Sample description

A random sample of 412 individuals aged at least 24 was selected for this study from 204 households in 8 villages from two towns. Of the 412 study participants, 364 were successfully interviewed, yielding a response rate of 88.3% (364/412). Of the 364 interviewed, 5 were excluded for suffering from mental retardation, and 359 participants provided complete data. After calculating the score for the subscale ‘lie’, 34 participants with a score of 60 or greater were further excluded, as this score indicated that the information that they provided may not have been credible. Therefore, 325 subjects were finally included in the analysis.

The data regarding socio-demographic characteristics, flood-related stressors, social support and personality traits are presented in Table 1 and the S1 Dataset. The sample was almost evenly distributed by gender and had a mean (SD) age of 57.79 (12.26) years. All the participants were Han ethnicity. Among all the respondents, 285 (87.7%) were married, 190 (58.5%) had attended primary school at most, 32 (9.8%) experienced at least three flood-related stressors, and 108 (33.2%) had a low level of social support. Furthermore, 118 (36.3%) had the trait of extraversion, 73 (22.5%) had the trait of tough-minded, and 87 (26.8%) had the trait of emotional instability.

**Table 1:** Characteristics of the participants (n = 325).

Variable	Values	Frequency	Percent (%)
Gender	Male	153	47.1
	Female	172	52.9
Marital status	Married	285	87.7
	Single/divorced/widowed	40	12.3
Age (years)	24–59	179	55.1
	60–87	146	44.9
Educational level	≤Primary school	190	58.5
	Junior middle school	104	32.0
	>Junior middle school	31	9.5
Flood-related stressors	≤3	293	90.2
	>3	32	9.8
Social support	High	81	24.9
	Medium	136	41.8
	Low	108	33.2
Extraversion	Middle	117	36.0
	Introversion	90	27.7
	Extraversion	118	36.3
Psychoticism	Middle	143	44.0
	Mild	109	33.5
	Tough-minded	73	22.5
Neuroticism	Middle	142	43.7
	Emotional stability	96	29.5
	Emotional instability	87	26.8

### Prevalence of PTSD and anxiety

The PCL-C total scores in this sample ranged from 17 to 62, with a mean (SD) total score of 25.05 (8.72). Based on a cut-off score of 38, the prevalence of PTSD 17 years after the flood was 9.5% (31/325). The mean (SD) total score of the SAS was 36.94 (7.92), and the total scores ranged from 20 to 57. Based on a cut-off score of 50, the prevalence of anxiety was 9.2% (30/325). In addition, 6.2% (20/325) of the participants suffered from both PTSD and anxiety, and 12.6% (41/325) suffered from either PTSD or anxiety. Furthermore, among the flood survivors who suffered from PTSD, 64.5% (20/31) also suffered from anxiety.

### Univariable analyses

The results of the univariable logistic regression analyses are presented in Table 2. According to these analyses, female sex (OR = 2.35, 95%CI = 1.05–5.27), experiencing at least three flood-related stressors (OR = 8.65, 95%CI = 3.69–20.31), having a low level of social support (OR = 5.55, 95%CI = 1.58–19.47), and having the trait of emotional instability (OR = 7.21, 95%CI = 2.78–18.72) were associated with PTSD, while experiencing at least three flood-related stressors (OR = 7.55, 95%CI = 3.18–17.94), having a low level of social support (OR = 7.38, 95%CI = 1.65–32.93), and having the trait of emotional instability (OR = 6.33, 95%CI = 2.42–16.59) were associated with anxiety.

**Table 2:** Univariable logistic regression analyses of the effects of socio-demographic characteristics, flood-related stressors, social support and personality traits on the odds of PTSD and anxiety.

Variable	Values	PTSD			Anxiety		
		Frequency (%)	OR (95% CI)	*P* value	Frequency (%)	OR (95% CI)	*P* value
Gender	Male	9(5.9)	1		9(5.9)	1	
	Female	22(12.8)	2.35(1.05–5.27)	0.039**	21(12.2)	2.23(0.99–5.02)	0.054*
Marital status	Married	28(9.8)	1		26(9.1)	1	
	Single/divorced/widowed	3(7.5)	0.74(0.22–2.57)	0.640	4(10.0)	1.11(0.37–3.36)	0.858
Age (years)	24–59	20(11.2)	1		17(9.5)	1	
	60–87	11(7.5)	0.65(0.30–1.40)	0.269	13(8.9)	0.93(0.44–1.99)	0.854
Educational level	≤Primary school	14(7.4)	1		15(7.9)	1	
	Junior middle school	12(11.5)	1.64(0.73–3.69)	0.232	10(9.6)	1.24(0.54–2.87)	0.614
	>Junior middle school	5(16.1)	2.42(0.80–7.27)	0.116*	5(16.1)	2.24(0.75–6.69)	0.147*
Flood-related stressors	≤3	19(6.5)	1		19(6.5)	1	
	>3	12(37.5)	8.65(3.69–20.31)	<0.001**	11(34.4)	7.55(3.18–17.94)	<0.001**
Social support	High	3(3.7)	1		2(2.5)	1	
	Medium	9(6.6)	1.84(0.48–7.01)	0.370	11(8.1)	3.48(0.75–16.10)	0.111*
	Low	19(17.6)	5.55(1.58–19.47)	0.007**	17(15.7)	7.38(1.65–32.93)	0.009**
Extraversion	Middle	11(9.4)	1		7(6.0)	1	
	Introversion	7(7.8)	0.81(0.30–2.19)	0.681	11(12.2)	2.19(0.81–5.89)	0.121*
	Extraversion	13(11.0)	1.19(0.51–2.78)	0.683	12(10.2)	1.78(0.68–4.69)	0.244
Psychoticism	Middle	18(12.6)	1		16(11.2)	1	
	Mild	10(9.2)	0.70(0.31–1.59)	0.395	10(9.2)	0.80(0.35–1.84)	0.603
	Tough-minded	3(4.1)	0.30(0.09–1.05)	0.059*	4(5.5)	0.46(0.15–1.43)	0.180*
Neuroticism	Middle	6(4.2)	1		6(4.2)	1	
	Emotional stability	4(4.2)	0.99(0.27–3.59)	0.982	5(5.2)	1.25(0.37–4.20)	0.724
	Emotional instability	21(24.1)	7.21(2.78–18.72)	<0.001**	19(21.8)	6.33(2.42–16.59)	<0.001**

*P ≤0.20

**P≤0.05

### Multivariable analyses

The results of the multivariable analyses are presented in Table 3. Factors with a *P* value less than or equal to 0.20 in the univariable logistic regression analyses were entered into the multivariable logistic regression analyses. The multivariable analyses indicated that female sex (OR = 3.34, 95%CI = 1.23–9.06), experiencing at least three flood-related stressors (OR = 9.48, 95%CI = 3.27–27.55), having a low level of social support (OR = 5.78, 95%CI = 1.35–24.71), and having the trait of emotional instability (OR = 6.82, 95%CI = 2.38–19.57) were risk factors for PTSD. Similarly, female sex (OR = 2.90, 95%CI = 1.12–7.53), experiencing at least three flood-related stressors (OR = 6.19, 95%CI = 2.27–19.93), having a low level of social support (OR = 6.81, 95%CI = 1.37–33.83), and having the trait of emotional instability (OR = 5.17, 95%CI = 1.84–14.57) were risk factors for anxiety.

**Table 3:** Multivariable logistic regression analyses of the factors associated with PTSD and anxiety in the univariable analyses.

Variable	Values	PTSD		Anxiety	
		OR (95% CI)	*P* value	OR (95% CI)	*P* value
Gender	Male	1		1	
	Female	3.34(1.23–9.06)	0.018*	2.90(1.12–7.53)	0.029*
Educational level	≤Primary school	1		1	
	Junior middle school	2.51(0.91–6.93)	0.075	1.85(0.68–5.02)	0.226
	>Junior middle school	3.30(0.84–13.02)	0.088	2.69(0.70–10.43)	0.152
Flood-related stressors	≤3	1		1	
	>3	9.48(3.27–27.55)	<0.001*	6.19(2.27–19.93)	<0.001*
Social support	High	1		1	
	Medium	1.57(0.35–7.15)	0.558	3.02(0.59–15.36)	0.183
	Low	5.78(1.35–24.71)	0.018*	6.81(1.37–33.83)	0.019*
Extraversion	Middle	N/A		1	
	Introversion	N/A	N/A	2.40(0.76–7.57)	0.136
	Extraversion	N/A	N/A	1.84(0.60–5.65)	0.287
Psychoticism	Middle	1		1	
	Mild	0.50(0.17–1.45)	0.200	0.67(0.24–1.89)	0.448
	Tough-minded	0.28(0.07–1.11)	0.070	0.43(0.12–1.53)	0.190
Neuroticism	Middle	1		1	
	Emotional stability	0.72(0.16–3.26)	0.668	1.01(0.26–3.98)	0.990
	Emotional instability	6.82(2.38–19.57)	<0.001*	5.17(1.84–14.57)	0.002*

N/A: Not applicable

*P≤0.05

## Discussion

Although it is well documented that PTSD and anxiety are the most prevalent psychological disorders that occur after natural disasters [3, 4, 46], the present study is one of the few to investigate the long-term psychological outcomes of flood survivors a long time after a devastating flood [47]. It is worth noting that the long-term psychological outcomes in this study were assessed 17 years after the flood. Currently, a study that assessed the long-term psychological outcomes of survivors 5 years after the 2007 UK flood [47] was considered to have conducted this type of assessment the longest time after a flood. Moreover, to the best of our knowledge, this is the first study to explore the prevalence of both PTSD and anxiety among survivors of the devastating 1998 Dongting Lake flood.

In particular, this study found that PTSD and anxiety were both prevalent among survivors 17 years after the 1998 Dongting Lake flood, and the prevalence of PTSD and anxiety was 9.5% and 9.2%, respectively. Collectively, 12.6% suffered from either PTSD or anxiety. These rates were much lower than those (22% for PTSD and 48% for anxiety) found among survivors of the 2007 summer floods in England 3 to 6 months after the flood [8]. However, the prevalence of PTSD reported in this study was much higher than that reported in some previous studies, which indicated that the estimated prevalence of PTSD two years after the 1998 Dongting Lake flood ranged from 8.6% to 9.2% [25, 26]. Flood severity and follow-up time might have greatly contributed to the variations in the prevalence observed in these studies. It is well documented that the prevalence of PTSD and anxiety decreases with time after a traumatic event [48]. Thus, this could explain why the prevalence of PTSD and anxiety in this study was lower than that observed in similar studies with smaller time gap for identifying PTSD and anxiety. However, the reason for the higher prevalence of PTSD found in this study than in the two previous studies estimating the prevalence of PTSD 2 years after the 1998 Dongting Lake flood could be that unlike those studies, this study was conducted in hard-hit areas, where survivors might have been exposed to trauma of a higher magnitude.

Additionally, this study identified the risk factors for PTSD and anxiety. Among the socio-demographic variables of interest, it was found that females were more likely to suffer from PTSD and anxiety than males when both were exposed to the flood. This finding is consistent with many previous studies, which found that females were more susceptible to certain psychiatric disorders after traumatic events [49–51]. However, similar to some studies [46, 52], this study did not find a significant relationship between PTSD or anxiety and age, education level and marital status, although some studies found that older age, having a low education level and being single/divorced/widowed were risk factors for PTSD and anxiety [53, 54]. It is therefore recommended to conduct additional similar studies with larger sample sizes to identify these relationships.

In addition, the results of the multivariable analyses in this study indicated that flood-related stressors were associated with PTSD and anxiety and that the likelihood of PTSD and anxiety was marginally significantly higher for participants who experienced at least three flood-related stressors. This finding is in line with many previous studies, which established that the intensity of exposure to a disaster is among the most robust predictive factors of psychiatric disorders [5, 55]. For example, Hashemian et al found that compared with those who were exposed to low-intensity warfare, individuals exposed to both high-intensity warfare and chemical weapons were at an 18.6 times higher risk of lifetime PTSD and a 14.6 times higher risk of anxiety [56]. Additionally, Zhang et al reported that compared with individuals who had not lost their relatives in the 2008 Wenchuan earthquake, those who had lost their relatives were at a 4.48 times higher risk of PTSD and a 4.16 times higher risk of anxiety [5].

Moreover, a protective effect of high levels of social support for PTSD and anxiety was found in this study. Individuals with low levels of social support were more likely to suffer from PTSD and anxiety when compared with those who had high levels of social support. This finding is consistent with many previous studies [16, 18]. For example, Udwin et al found that social support was significantly associated with PTSD among young adults who survived a shipping disaster [16]. Additionally, in a study by Basoglu et al, it was found that a lack of social support predicted anxiety symptoms among survivors of torture in Turkey [18]. It is well established that social support can be considered a buffer when stress occurs [57] and thus plays an important role in the development of long-term psychological outcomes [58].

The results of this study also indicated that the trait of emotional instability was a risk factor for both PTSD and anxiety. Personality traits are traditionally conceptualized as dimensions of individual differences in tendencies to show consistent patterns of feelings, thoughts, and actions across developmental periods and contexts [59]. The interface between personality and psychopathology is well established, and the trait of neuroticism has been reported to be a predictor of many psychological disorders, including PTSD and anxiety [60, 61]. In this study, participants with emotional instability, indicating a high level of neuroticism, were at a higher risk of suffering from PTSD and anxiety a long time after the flood. This finding is in agreement with a recent meta-analysis which indicated that the trait of neuroticism was a risk factor for PTSD [62].

It is noteworthy to indicate here that this study found that among the flood survivors who suffered from PTSD, 64.5% also suffered from anxiety and the risk factors for PTSD and anxiety were the same, namely female sex, experiencing at least three flood-related stressors, having a low level of social support and having the trait of emotional instability. Previous studies indicated that although PTSD and anxiety are independent psychiatric disorders, they share many common risk factors in trauma-related populations [49]. For example, Kolltveit et al found that female sex, older age and more serious exposure to war stressors were risk factors for not only PTSD but also anxiety in a sample of adolescents who were exposed to war stressors in Gaza [53]. Furthermore, in a study by Groome et al, PTSD and anxiety symptoms were both more severe in a sample of children survivors of the 1999 Greek earthquake who were female, lived in much more damaged district and were exposed to a higher level of threat [3]. One possible explanation for the similar risk factors for PTSD and anxiety observed in these studies, including this one, could be the frequently observed high occurrence of anxiety among PTSD patients in trauma-related populations, which may lead to a combined stress model with shared vulnerability [49, 63].

Some limitations of this study should be acknowledged. Firstly, although this survey was managed by well-trained investigators, the survey itself was essentially retrospective. For example, 17 years after the 1998 Dongting Lake flood, the intensity of exposure to the flood measured in this study may not have always been accurate; however, the result, demonstrating that the intensity of exposure to the flood was associated with both PTSD and anxiety, was consistent with many previous studies [5, 55]. Secondly, the diagnoses of PTSD and anxiety were determined according to self-report questionnaires rather than structured clinical interviews, and this approach may have led to a higher estimation of the prevalence of PTSD and anxiety. Thirdly, the PCL-C used in this study was developed according to DSM-IV criteria. Thus, the symptom of negative alterations in cognition and mood was not included in this study, as this is a newly added symptom cluster in the DSM-V [64]. Fourthly, income, which is considered an important indicator of socioeconomic status, was not analyzed in this study because most of the participants did not want to provide information related to their income. Additionally, all participants enrolled in this study were Han ethnicity, and for this reason, the results may not be applicable to other populations. Finally, participants enrolled in this study were from hard-hit areas of the flood. Thus, it may not be appropriate to generalize the findings in the present study to other flood-affected regions with populations exposed to different degrees of flood intensity.

Despite these limitations, the results of this study have several strengths and implications. Firstly, it was found that nearly an eighth of the Dongting Lake flood survivors still suffered from either PTSD or anxiety 17 years after the flood. This finding indicates that floods can have a long-term adverse psychological impact on survivors, which strongly agrees with the fact that natural disasters can have long-lasting effects on survivors’ mental health [1, 2]. Secondly, the potentially associated variables measured in this study included not only socio-demographic variables and trauma-related stressors but also social support and personality traits, which may play important roles in the incidence and prognosis of psychological disorders [27, 62]. Last but not least, the results of this study emphasized the importance of implementing early and effective psychological interventions among flood survivors, including cognitive behavioral therapy, which has been reported to be an effective intervention for treating PTSD and DSM-V anxiety disorders [65, 66].

In conclusion, this cross-sectional study explored the long-term psychological outcomes of flood disaster survivors from hard-hit areas of the 1998 Dongting Lake flood and found that PTSD and anxiety were both prevalent in this population. Moreover, female sex, experiencing at least three flood-related stressors, having a low level of social support and having the trait of emotional instability were risk factors for developing PTSD and anxiety a long period of time after the flood. More longitudinal studies with larger sample sizes are needed to examine the dynamic changes in the long-term psychological outcomes of flood survivors and to further clarify how the associated factors interact to contribute to these outcomes, as well as the direct and indirect effects of the associated factors.

## Supporting information

S1 Dataset The original data of the investigation. (SAV)
